# Binding Folate Receptor Alpha Autoantibody Is a Biomarker for Leucovorin Treatment Response in Autism Spectrum Disorder

**DOI:** 10.3390/jpm14010062

**Published:** 2024-01-01

**Authors:** Richard E. Frye, Patrick J. McCarty, Brianna A. Werner, Adrienne C. Scheck, Heidi L. Collins, Steven J. Adelman, Daniel A. Rossignol, Edward V. Quadros

**Affiliations:** 1 Rossignol Medical Center, Phoenix, AZ 85050, USA; 2 Autism Discovery and Treatment Foundation, Phoenix, AZ 85050, USA; rossignolmd@gmail.com; 3 Tulane University Medical School, New Orleans, LA 70112, USA; pmccarty2@tulane.edu; 4 Creighton University School of Medicine, Phoenix, AZ 85013, USA; briannawerner@creighton.edu; 5 Department of Child Health, University of Arizona College of Medicine—Phoenix, Phoenix, AZ 85004, USA; ascheck@arizona.edu; 6 Vascular Strategies LLC, Plymouth Meeting, PA 19462, USA; hcollins@vascularstrategy.com (H.L.C.); sadelman@vascularstrategy.com (S.J.A.); 7 Rossignol Medical Center, Aliso Viejo, CA 92656, USA; 8 Department of Medicine, State University of New York—Downstate, Brooklyn, NY 11203, USA; edward.quadros@downstate.edu

**Keywords:** autism spectrum disorder, folate receptor alpha, soluble folate binding proteins, folinic acid, leucovorin

## Abstract

Autism spectrum disorder (ASD) affects up to 1 in 36 children in the United States. It is a heterogeneous neurodevelopmental disorder with life-long consequences. Patients with ASD and folate pathway abnormalities have demonstrated improved symptoms after treatment with leucovorin (folinic acid), a reduced form of folate. However, biomarkers for treatment response have not been well investigated and clinical trials are lacking. In this retrospective analysis, a cohort of prospectively collected data from 110 consecutive ASD clinic patients [mean (SD) age: 10.5 (6.2) years; 74% male] was examined. These patients all underwent testing for folate receptor alpha autoantibodies (FRAAs) and soluble folate binding proteins (sFBPs) biomarkers and were treated with leucovorin, if appropriate. Analyses examined whether these biomarkers could predict response to leucovorin treatment as well as the severity of ASD characteristics at baseline. The social responsiveness scale (SRS), a measure of core ASD symptoms, and the aberrant behavior checklist (ABC), a measure of disruptive behavior, were collected at each clinic visit. Those positive for sFBPs had more severe ASD symptoms, and higher binding FRAA titers were associated with greater ABC irritability. Treatment with leucovorin improved most SRS subscales with higher binding FRAA titers associated with greater response. Leucovorin treatment also improved ABC irritability. These results confirm and expand on previous studies, underscore the need for biomarkers to guide treatment of folate pathways in ASD, and suggest that leucovorin may be effective for children with ASD.

## 1. Introduction

One in 36 children in the United States are diagnosed with autism spectrum disorder (ASD) as estimated by the Autism and Developmental Disabilities Monitoring Network, with a continuing increase in prevalence [1]. Although the diagnosis of ASD is defined by behavioral features, both brain-based and systematic biological abnormalities are associated with ASD [2]. Brain-based abnormalities include abnormalities in monoamine neurotransmitter production, imbalances in the excitatory to inhibitory balance, and epilepsy [3,4,5]. Systemic abnormalities including immune dysfunction, oxidative stress and metabolic disorders may also be linked with ASD [6,7,8,9,10]. These abnormalities can interact to negatively reinforce each other. Metabolic disorders are particularly compelling since many such disorders can be mitigated with safe, well-tolerated treatments [9,10].

One of the compelling metabolic abnormalities associated with ASD involves central folate metabolism. It appears that some children with ASD have difficulty with transporting folate into the central nervous system (CNS). The severe form of this disorder, cerebral folate deficiency (CFD), is diagnosed by having a below-normal concentration of folate in the cerebral spinal fluid (CSF) (which requires an invasive lumbar puncture to measure) with a normal folate concentration in the blood [11]. The primary transporter of folate into the CNS is the folate receptor α (FRα). In CFD, the FRα becomes less functional for one of several reasons. One of the major causes of disrupted FRα function is the presence of one of two autoantibodies that bind to the receptor protein, known as the blocking and binding FRα autoantibodies (FRAAs). FRAAs bind to the FRα and block the binding of folate to the FRα or interfere with its function [12]. Recently, the significance of a third class of proteins known as soluble folate binding proteins (sFBPs) has also been linked to central folate abnormalities [13]. Other major causes of FRα dysfunction includes mitochondrial disorders [14]. Although genetic disorders of the FRα have been described, they remain rather rare.

CFD appears to be linked to neurodevelopmental and psychiatric disorders. CFD was associated with ASD soon after it was first described [15], and further studies suggested that it may affect a high number of children with ASD [16,17,18]. In addition, CFD has also been associated with inflammatory conditions, such as juvenile rheumatoid arthritis [19].

One important aspect of CFD is that it can be treated with a specific reduced form of folate (leucovorin also known as folinic acid), and treatment can significantly improve neurological and neurodevelopmental symptoms [20]. This is also true for ASD, based on controlled clinical trials that have been performed [20]. Preliminary findings also suggest that symptoms of depression, suicidal ideations, and schizophrenia can respond to such treatment [21].

Since the gold standard for diagnosing CFD requires a lumbar puncture, which is an invasive procedure, there is great interest in biomarkers of CFD that can be measured without a lumbar puncture. The FRAAs predicted response to leucovorin in a controlled clinical trial [22], and sFBPs appear to suggest a more severe form of leucovorin-responsive ASD [13]. However, beyond the presence or absence of these blood-based biomarkers, biomarkers titers have not been shown to be useful in predicting response to leucovorin treatment. Furthermore, many ASD patients are on other medications or supplements that can contribute to the expected effect of leucovorin. To further determine whether FRAAs or sFBPs can be used to predict response to leucovorin and to examine the effect of other concurrent supplements, we performed a longitudinal study of a large cohort of children with ASD, both treated and not treated with leucovorin, in a clinical setting. The clinical setting in which they were seen systematically measures the severity of ASD symptoms and behaviors at each visit, making a longitudinal study possible.

## 2. Materials and Methods

The Phoenix Children’s Hospital (Phoenix, AZ, USA) Neurodevelopmental Disorders program recruited participants from a natural history study on ASD. Clinical patients were provided the opportunity to contribute their clinical data to the study. The Institutional Review Board at Phoenix Children’s Hospital approved the protocol. Parents of participants provided written informed consent.

The ASD diagnosis was verified in one of two ways. Patients could have completed either an Autism Diagnostic Interview-Revised (ADI-R; WPS, Torrance, CA, USA) or Autism Diagnostic Observation Schedule (WPS, Torrance, CA, USA), the gold-standard diagnostic instruments. Alternatively, a combination of tools could be used to verify their diagnosis. These included documentation of Diagnostic Statistical Manual of Mental Disorders Fifth Edition criteria, documentation of functional limitations, a Social Responsiveness Scale (SRS; WPS, Torrance, CA, USA), score with the ASD range and verification of the diagnosis by the Principal Investigator (R.E.F.), who is a specialist in the diagnosis and treatment of ASD. The SRS correlates well with gold-standard diagnostic instruments and is standardized and validated [23,24]. This latter method for diagnosis has been validated in our previous study by evaluating participants diagnosed with this latter method using the ADI-R. We found that their scores were well within the ASD diagnostic range [22].

As part of the standard clinic intake and follow-up processes, patients completed a standardized medical history questionnaire, the SRS (represented as t-scores), and the Aberrant Behavior Checklist (ABC; represented as raw scores; Slosson Educational Publications, East Aurora, NY, USA). The SRS and ABC were repeated at each visit to follow the changes in ASD-related symptoms with treatment during clinical care. This standardized prospective collection of outcome data to study treatment effectiveness has been used in our previous studies [25,26]. As this data was derived from clinical practice, all decision-making and prescribing was part of standard medical care, and leucovorin treatment was prescribed using standard guidelines [20].

Only consented participants who had previously had FRAAs testing performed were included in this study. Clinical information was abstracted from the patient’s chart at each visit, including stopping and starting medication and SRS and ABC scores. Age was calculated at each visit so that personal health identifiers, such as dates, could be removed to deidentify the data. Thus, the final dataset was analyzed under 45 CFR 46 exemption 4.

### 2.1. FRAA Assay

The Vascular Strategies (Plymouth Meeting, PA, USA) performed the FRAA in their CLIA licensed laboratory. Blocking FRAAs were measured using an in vitro functional assay. Binding IgG measured binding FRAAs using an enzyme-linked immunosorbent assay (ELISA) [27]. A higher-than-expected binding of ^3^H-folic acid in the blocking assay signified the presence of sFBPs [13].

### 2.2. Statistical Analysis

To analyze the difference in patient characteristics across FRAA assay biomarkers, Pearson chi-square test was calculated on cross tabulation tables. For age, a multivariate linear model was used. Differences in behavior measures across the patient characteristics of age, developmental profile, sex, and presence of the binding and blocking FRAAs and/or sFBPs were compared using multivariate linear models for each SRS and ABC subtest separately. An alpha of 5% was used as a cutoff for significance.

To analyze the effect of leucovorin treatment, we used a mixed-model with random effects of subject and time to control for repeated effects of subject level mean and variance with an autoregressive moving average covariance structure. Leucovorin was given to the population in two ways: orally and as part of a subcutaneous (SQ) B12 injection. Thus, a dichotomous variable to represent whether the individual was on leucovorin vs. whether they were not on leucovorin was derived. To determine the effect of leucovorin treatment on changes in SRS and ABC scores, each individual score was used as the dependent variable in separate mixed-models. Independent variables included descriptive characteristics of age (in days) and sex, time from first seen in clinic (in days), and the effects of other treatments which might affect language, including B12, carnitine, fatty acids, stimulants, anti-psychotics, and beta-blockers. Since one of the goals of this study is to understand whether FRAA assay biomarkers can be used to predict response to therapy, the interaction between these biomarkers and treatment with leucovorin was included in the model. The final model was simplified to only significant variables and variables that were dependent on significant interactions. An alpha of 5% was used as a cutoff for significance.

To highlight the effects of leucovorin on significant outcomes measures, the coefficients of the linear equations estimated in the models were used to produce a graph.

## 3. Results

A description of the characteristics of the 110 patients who underwent FRAA testing is first provided, followed by an analysis of the change in ASD and related symptoms with leucovorin treatment.

### 3.1. Folate Biomarkers

A total of 13% of participants were sFBPs positive, 65% were binding FRAA positive, 4% were blocking FRAA positive and 29% were negative for FRAAs or sFBPs (Table 1). Half of the patients with sFBPs were also positive for binding FRAAs with a titer that was significantly lower [F(1,109) = 4.75, *p* = 0.03] than those without sFBPs. Those positive for the blocking FRAA demonstrated significantly higher binding FRAA titers [F(1,109) = 5.59, *p* = 0.02] than those without the blocking FRAA.

### 3.2. Patient Characteristics

Characteristics of patients positive for sFPBs and/or blocking and/or binding FRAAs as well as those negative for all folate biomarkers are given in Table 2. Most characteristics were similar across groups. Those with sFBPs and/or binding FRAAs were more likely to be treated with oral leucovorin while those negative for all folate biomarkers were less likely to be treated with oral leucovorin. Those with sFBPs were less likely to be treated with alpha-adrenergic medications, while those negative for all folate biomarkers were likely to be using herbal medication.

Age, developmental profile and binding and blocking FRAAs were not significant for any SRS scale, so the model was simplified to include sex and sFPBs as independent variables and binding and blocking FRAA titers as covariates.

Most SRS scales were from 5 to 7 points higher (more severe ASD symptoms) for females. Specially, females demonstrated worse Awareness (β = 7.2, F(1,99) = 7.99, *p* < 0.01), Cognition (β = 5.4, F(1,99) = 7.41, *p* < 0.01), Communication (β = 7.9, F(1,99) = 13.02, *p* < 0.001), Mannerisms (β = 6.0, F(1,99) = 5.39, *p* < 0.05) and Total (β = 7.2, F(1,99) = 11.39, *p* = 0.001) SRS scores.

Most SRS scales were from 5 to 7 points higher (more severe ASD symptoms) for those with sFBPs. Specifically, those with sFPBs in their blood demonstrated worse Awareness (β = 7.2, F(1,99) = 4.68, *p* < 0.05), Cognition (β = 6.7, F(1,99) = 6.47, *p* = 0.01), Motivation (β = 7.4, F(1,99) = 4.98, *p* < 0.05) and Total (β = 5.8, F(1,99) = 4.23, *p* < 0.05) SRS scores.

Those with sFPBs in their blood demonstrated higher Social Withdrawal (β = 6.4, F(1,108) = 7.19, *p* < 0.01), and older individuals demonstrated lower Hyperactivity scores (β = −0.002/day of age, F(1,108) = 12.93, *p* < 0.001).

### 3.3. Effectiveness of Leucovorin Treatment

In general, mixed-models did not find any effect of developmental trajectory, sex, or age on dependent variables, so these characteristics are not discussed further.

#### 3.3.1. Social Responsiveness Scale

**Figure 1:**
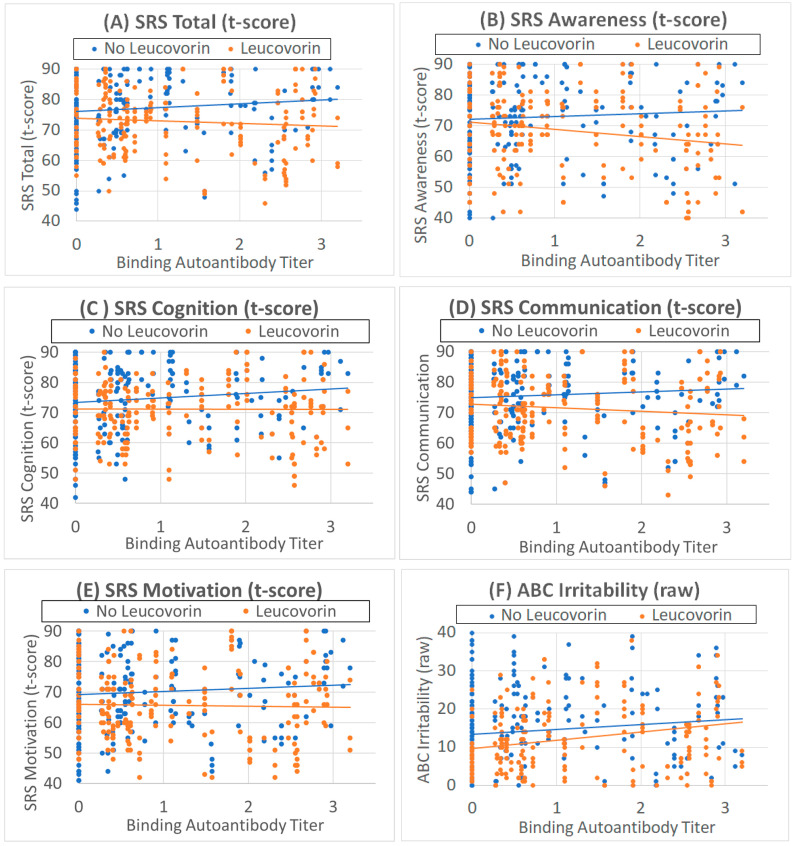
Behavioral scores are related to binding folate receptor alpha autoantibody titers. (**A**–**E**) Social Responsiveness Scale (SRS) t-scores are better (lower scores) as binding folate receptor alpha autoantibody titers increase with leucovorin treatment (orange points and line), while SRS scores are worse (higher scores) as binding folate receptor alpha autoantibody titers increase without leucovorin treatment (blue points and line). (**F**) Aberrant behavior checklist Irritability scores worsen as binding folate receptor alpha autoantibody titers increase, with leucovorin overall improving irritability.

The effect of leucovorin on the total SRS score was affected by the binding FRAA titer [F(1,395.4) = 5.30, *p* = 0.02] such that the SRS Total score worsened by 1.6 (0.70) for those not on leucovorin and improved by 0.88 (0.96) for each 1 unit of binding titer (Figure 1A). Subcutaneous B12 improved and beta-blockers worsened the SRS Total score (Table 3).

The effect of leucovorin on the SRS Awareness score was affected by the binding FRAA titer [F(1,435.8) = 6.18, *p* = 0.01] such that the Awareness worsened by 2.5 (1.03) for those not on leucovorin and improved by 2.48 (1.12) for each 1 unit of binding titer (Figure 1B). Fatty acids also improved Awareness (Table 3).

The effect of leucovorin on the SRS Cognition score was affected by the binding FRAA titer [F(1,417.0) = 4.80, *p* = 0.03] such that the Cognition worsened by 1.8 (0.82) for those not on leucovorin and improved by 0.86 (0.90) for each 1 unit of binding titer (Figure 1C). Fatty acids and SQ B12 improved and beta-blockers worsened SRS Cognition (Table 3).

The effect of leucovorin on the SRS Communication score was affected by the binding FRAA titer [F(1,380.1) = 4.62, *p* = 0.03] such that the Communication SRS score worsened by 1.6 (0.75) for those not on leucovorin and improved by 1.2 (1.00) for each 1 unit of binding titer (Figure 1D). Fatty acids and SQ B12 improved SRS Communication (Table 3).

The effect of leucovorin on the SRS Motivation score was affected by the binding FRAA titer [F(1,411.9) = 3.91, *p* = 0.05] such that the Motivation score worsened by 1.7 (0.87) for those not on leucovorin and improved by 1.0 (1.11) for each 1 unit of binding titer (Figure 1E). Fatty acids and SQ B12 improved SRS Motivation (Table 3).

Leucovorin did not have a significant effect on the SRS Mannerisms score, but SQ B12 improved and beta-blockers worsened SRS Mannerisms (Table 3).

#### 3.3.2. Aberrant Behavior Checklist

Leucovorin treatment decreased ABC Irritability [F(1,434.2) = 9.673, *p* < 0.01] by 2.4 (0.76) points. Irritability increased as binding FRAA titers increased [F(1,109) = 3.78, *p* = 0.05] such that Irritability increased by 1.5 (0.80) points for each unit increase in the binding FRAA titer (Figure 1F). Beta-blockers worsened ABC Irritability (Table 4).

ABC social Withdrawal and Stereotypy scales were improved by fatty acid supplementation, and Hyperactivity was improved by carnitine (Table 4).

### 3.4. Effect of Leucovorin on Outcomes as a Function of Binding Titers

The outcomes that were significantly related to the binding titer were graphed as a function of the binding titer in order to better display the magnitude of the effect. The coefficients from the statistical model were used to produce the graph. The equation accounted for the fixed effect of leucovorin treatment on scores, the effect of binding titer on score and the effect of binding titer on score with leucovorin treatment.

**Figure 2:**
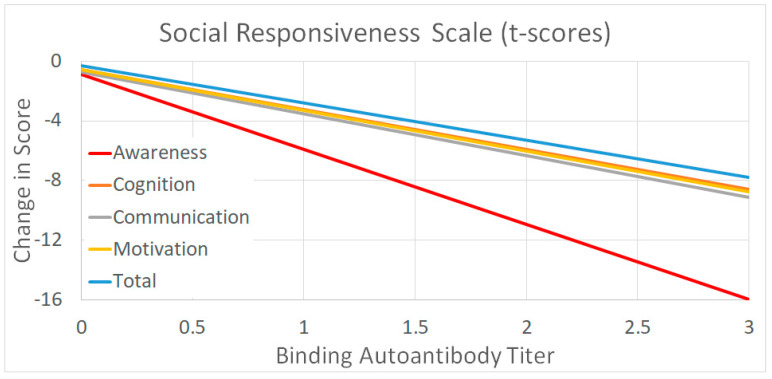
The average response to leucovorin as a function of binding titers for the outcomes that were significantly related to the binding titer.

Figure 2 demonstrates that the magnitude of the effect is dependent on the binding autoantibody titer with the relation varying in magnitude depending on the outcome measure. Clearly leucovorin influences SRS Awareness the most with an individual having a binding autoantibody of 3.0 demonstrating, on average, a 16-point drop in their SRS score. This is clearly enough to change the category of severity on the SRS from severe to moderate, moderate to mild, or mild to typically developing. The other SRS scales demonstrated a more moderate effect, decreasing the SRS score by 8 points for an individual with a binding titer of 3.0.

## 4. Discussion

We reviewed 110 patients with ASD who were tested for FRAAs and sFBPs to guide clinical management by identifying the clinical characteristics associated with these biomarkers and to determine whether the biomarkers could predict treatment response. Overall, female sex and testing positive for sFBPs were associated with worse scores on the SRS, suggesting that these were more severely affected patients. For most of the scales of the SRS, change in the scores when treated with leucovorin was dependent on the binding FRAA titer such that higher titers were associated with more severe ASD symptoms when not treated and less severe ASD symptoms when treated with leucovorin. For the ABC scales, leucovorin only improved Irritability, but this treatment response was not related to folate biomarkers. Interestingly, higher binding FRAA titers were associated with worse Irritability. Both mB12 SQ and fatty acids were associated with better SRS scores on a few subscales, fatty acids were associated with better ABC scores on two subscales and carnitine was associated with a better score on ABC Hyperactivity. In contrast, beta-blockers were associated with worse scores on most SRS subscales and ABC Irritability. These findings will be discussed in detail below.

Our recent study reported the first series of patients with ASD and sFBPs [13]. Cancer research has studied sFBPs [28], but our recent paper was the first time it was studied in neurodevelopmental disorders. The patients with sFBPs appeared to be more symptomatic and more complex than other ASD patients, but no quantitative measurement of severity was used to characterize the cohort. This study demonstrates that patients with sFBPs have more severe ASD symptoms as compared to those without sFBPs by about 5–6 points on the SRS scale, an amount that could easily push them into a higher level of ASD severity, thereby confirming the qualitative observation from our previous study [13]. We also found that sFBPs status was not a biomarker for treatment response. However, in our previous study, patients with sFBPs required more aggressive leucovorin treatment, requiring leucovorin doses about twice the typically prescribed doses to obtain a therapeutic effect. Thus, it is possible that the negative biomarker result for sFBPs in this study was due to under dosing patients in this subgroup. Further studies are needed to determine if sFBPs status might be a biomarker of leucovorin dosing.

This study found that the binding FRAA predicted response to leucovorin treatment for 5 of the 6 SRS scales. The SRS measures ASD symptoms, suggesting that ASD symptom improvement with leucovorin treatment is related to the binding FRAA titers. This is due to two opposing effects. It appears that those with higher binding FRAAs that were not treated with leucovorin had more severe ASD symptoms than those with low binding FRAA titers. In contrast, those with higher FRAA titers who were treated with leucovorin appear to have less severe ASD symptoms than those that were treated and had lower binding FRAA titers. Thus, overall, those with high FRAA titers had a greater response to leucovorin both because they were relatively more severe without treatment and relatively less severe with treatment. As seen in Figure 2, those with some of the highest binding FRAA titers can have, on average, a 8 to 16 point response on SRS subscales, an amount that can move them into more favorable severity categories.

The effect of FRAAs on ASD severity has been investigated [29,30,31,32]. One study found that those with blocking FRAAs had more favorable development and metabolic profiles than those with binding FRAAs, and those with binding FRAAs had higher B12 concentrations in the blood than those with blocking FRAAs [33]. Another study in ASD children found that FRAA positivity was associated with significantly lower developmental and language scores as compared to those that were FRAA negative [12]. Lastly, a double-blind clinical trial found that being FRAA positive was associated with significantly better response to leucovorin treatment. These previous studies did not examine titers but rather being FRAA positive or negative, so although they are not absolutely comparable with the analysis in this study, their findings support and are consistent with our findings.

The binding FRAA titer was found to be associated with increased Irritability, and treatment with leucovorin improved Irritability. A previous double blind controlled trial found that leucovorin treatment improved Irritability in ASD [25]. This relationship with Irritability may be important, as the medications indicated for Irritability in ASD are anti-psychotic medications which can have both short-term and long-term adverse effects [34].

Previous controlled studies have demonstrated that leucovorin can improve verbal communication and behavior [25,34] as well as core ASD symptoms [31], and a recent meta-analysis of 21 studies using leucovorin alone or in combination with other treatments has confirmed these findings [20]. There are few treatments that improve symptoms of ASD, and improvements in the SRS in this study support the notion that leucovorin improves core ASD symptoms. Leucovorin is an extremely safe and well-tolerated treatment, which is important in this vulnerable population [20].

Few validated biomarkers are available to predict treatment response or assist with diagnosis in ASD. Studies have suggested that biomarkers of folate one-carbon metabolism are very promising to assist with ASD diagnosis and FRAAs are promising biomarkers to predict treatment response [35]. This research as well as this study highlights the significance of the folate pathway in ASD and promises to further our understanding of the underlying biological abnormalities associated with ASD.

This study also found that several other treatments influenced core ASD symptoms as well as disruptive behaviors. Omega 3 fatty acids improved SRS social Awareness, Cognition, Communication, and Motivation as well as ABC Social Withdrawal and Stereotypy. These results are consistent with a recent moderate size double blind placebo-controlled (DBPC) study on omega 3 fatty acid supplementation in ASD in which improvements were seen in social communication, stereotyped behavior, and core symptoms [36]. Significant improvements in the SRS with omega 3 fatty acids were also seen in an open label study [37]. Furthermore, a meta-analysis of randomized controlled trials studying omega 3 fatty acids found improvements on ABC Social Withdrawal and Stereotypy subscales consistent with our study [38].

Methyl-cobalamin (mB12) was found to improve the SRS Total score, as well as all subscales except SRS Awareness, in this study. A recent meta-analysis found that clinical studies support the positive effect of cobalamin, particularly subcutaneously injected mB12, on ASD symptoms [39]. One small prospective uncontrolled study found that mB12 improved core symptoms of ASD as well as the developmental quotient [40] while another medium size prospective uncontrolled study found that subcutaneously injected mB12 improve development as well as biochemical markers of transsulfuration [41]. A randomized DBPC study found that subcutaneously injected mB12 improved the Clinical Global Scale Impression score [42]. Thus, our study supports the growing literature on the positive effects of B12 in ASD.

Carnitine was found to improve ABC Hyperactivity in this study. Carnitine has been found to improve core ASD symptoms in two DBPC studies [43,44] and ABC Hyperactivity when combined with risperidone [45]. Carnitine may be particularly important to supplement in individuals with ASD since, as a group, carnitine is low in many children with ASD [46,47] and a subset of children with ASD may have a genetic deficiency in carnitine production [45,47,48].

Beta-blockers were found to increase ABC Irritability in this study. This is somewhat contrary to the literature as beta-blockers have been found to improve verbal problem solving [49] and word fluency [49], and nonverbal communication [50,51], and regulate brain networks to improve information processing [52] in controlled studies and has recently been reported to improve challenging behaviors at high doses [53]. Although this would seem paradoxical, it may be due to the patient population in which beta-blockers were prescribed had more severe behaviors at baseline as beta-blockers are typically used in an attempt to improve challenging behaviors.

This work has several limitations, most notably the open-label methodology. A study derived from clinical data does provide insight into the effectiveness of a treatment in the clinic but also opens the study to biases in clinical practice. The analysis we used attempted to control confounding factors such as concurrent medication use, but controlled clinical trials will be necessary to confirm the findings of this study.

## 5. Conclusions

We evaluated a cohort of ASD patients assessed in the clinic for abnormalities in folate metabolism and treated per routine clinical practice. Overall, leucovorin treatment improved core ASD features in this cohort, and the binding FRAA data was useful in determining which patients might have the greatest response to treatment. Verification of these findings will require larger controlled studies; however, our findings may be an important starting point for the treatment of individuals with ASD and demonstrate the effectiveness of leucovorin in the clinical setting.

## Figures and Tables

**Table 1:** Folate biomarker prevalence and titers across folate biomarker groups (* *p* < 0.05; sFBPs = soluble folate binding proteins).

	Folate Biomarker Groups			
	sFBPs+ (n = 14)	Blocking+ (n = 4)	Binding+ (n = 70)	Overall
sFBP Prevelence	100%		10%	13%
Blocking Prevelence		100%	6%	4%
Blocking Titer (pmol/mL)		2.45 (0.65)	0.14 (0.59)	0.09 (0.47)
Binding Prevelence	50%	100%	100%	65%
Binding Titer (pmol/mL)	0.34 (0.40) *	2.03 (1.09) *	1.37 (0.96)	0.88 (1.01)

**Table 2:** Characteristics of patients by folate biomarker (* *p* < 0.05; ** *p* ≤ 0.01 *** *p* ≤ 0.001 sFBPs = soluble folate binding proteins).

	sFBPs+ (n = 14)	Blocking+ (n = 4)	Binding+ (n = 70)	Negative (n = 32)	Overall (n = 110) †
Age (SD) in years	11.0 (5.1)	8.2 (4.2)	10.5 (5.8)	10.1 (7.2)	10.5 (6.2)
Sex (% Male)	93%	75%	76%	63%	74%
Race: White/Black/Asian	71%/29%/0%	50%/25%/25%	80%/3%/10%	***85%/0%/13%*** *	81%/5%/10%
Ethnicity (% Hispanic)	7%	25%	4%	16%	8%
Development: Birth/Plateau/Regression	0%/36%/64%	0%/0%/100%	11%/37%/51%	3%/53%/41%	8%/42%/49%
Treatments					
Oral Leucovorin/Other Oral Folate	***86%*** */64%	75%/75%	***70%*** ***/48%	***22%*** ***/44%	57%/47%
mB12 & Leucovorin SQ/Oral B12	43%/36%	75%/50%	31%/41%	25%/34%	30%/38%
Other Bs/Non B Vits	21%/43%	75%/50%	32%/56%	31%/53%	31%/53%
ASD MVI/Other MVI	14%/29%	50%/25%	25%/27%	28%/32%	25%/29%
Antioxidants/Minerals	29%/35%	50%/50%	30%/37%	31%/44%	29%/36%
Carnitine/CoQ 10	36%/21%	75%/50%	41%/39%	50%/34%	42%/36%
Melatonin/Herbal Meds	14%/0%	0%/0%	18%/4%	22%/***19%*** *	18%/8%
Fatty Acids/Amino Acids	29%/21%	50%/0%	31%/18%	25%/13%	29%/16%
Stimulants/Alpha Adenergics	14%/***0%*** *	0%/0%	16%/23%	9%/28%	14%/23%
Allergy & Asthma/GI Meds	29%/43%	25%/75%	34%/59%	38%/59%	34%/57%
Inflamatory & Immune/Anti-Microbials	7%/7%	0%/25%	13%/14%	9%/15%	12%/14%
Antipsychotics/Other Psychotropics	21%/7%	0%/50%	16%/9%	9%/13%	13%/10%
SSRIs/Beta-Blockers	21%/7%	25%/50%	18%/20%	13%/16%	16%/17%
Anti-Epileptic Meds/Diuretic	21%/0%	0%/25%	21%/4%	31%/3%	25%/4%
Thyroid Meds/Other Hormones	0%/7%	0%/0%	1%/4%	***13%*** **/3%	5%/5%

† Overall number of participants is less than the totals for the number of participants for each individual folate biomarker since some participants can be positive for more than one folate biomarker.

**Table 3:** Effect of concurrent treatments on Social Responsiveness Scale (* *p* < 0.05; ** *p* < 0.01; *** *p* < 0.001).

SRS Subscale	mB12 SQ	β Blocker	Fatty Acids
Total	−2.8 (1.1) **	2.8 (1.2) *	
Awareness			−3.0 (1.6) *
Cognition	−4.3 (1.3) ***	2.8 (1.4) *	−2.4 (1.2) *
Communication	−2.3 (1.2) *		−2.4 (1.2) *
Motivation	−3.0 (1.4) *		−3.0 (1.4) *
Mannerisms	−3.6 (1.1) ***	2.6 (1.3) *	

**Table 4:** Effect of concurrent treatments on Aberrant Behavior Checklist (* *p* < 0.05; ** *p* < 0.01; *** *p* < 0.001).

ABC Subscale	β Blocker	Fatty Acids	Carnitine
Irritability	4.8 (1.3) ***		
Social Withdrawal		−2.0 (0.9) *	
Stereotypy		−1.3 (0.5) **	
Hyperactivity			−2.2 (0.7) **

## Data Availability

The data presented in this study are available on request from the corresponding author.
